# Observing and Suppressing Metallization in MoS_2_ for Near-Ideal Spin Filtering

**DOI:** 10.1021/acsami.5c15955

**Published:** 2025-11-19

**Authors:** Ting-Chun Huang, Yu-Xin Chen, Yu-Lin Chen, Meng-Ting Wu, Chi-Feng Pai, Chiashain Chuang, Ya-Ping Hsieh, Mario Hofmann

**Affiliations:** † 71556 Institute of Atomic and Molecular Sciences, Academia Sinica, Taipei 115, Taiwan; ‡ Department of Physics, 33561 National Taiwan University, Taipei 106, Taiwan; § Department of Electronic Engineering, 34900 Chung Yuan Christian University, Taoyuan 320, Taiwan; ∥ Department of Materials Science and Engineering, 63393 National Taiwan University, Taipei 10617, Taiwan

**Keywords:** magnetic tunnel junction, spin filter, magnetoresistance, MoS_2_, metalization

## Abstract

Two-dimensional (2D) transition-metal dichalcogenides (TMDCs) offer band alignments that are well-suited for minority-spin filtering when interfaced with ferromagnets. Unfortunately, interfacial hybridization results in the emergence of metallic states in MoS_2_ that have limited previous results significantly below theoretical expectations. Here, we study the metallization at the Co/MoS_2_ interface and devise a method to overcome its detrimental impact on spin transport. Using a contamination-free fabrication process, clear evidence of hybridization was observed, which increases the magnetic anisotropy of the interface. Magneto-transport and theoretical analysis reveal that the metallic wave functions extend into the MoS_2_ layer, suppressing its ability as a filter barrier. The spin-filtering ability can be restored when separating the MoS_2_ by only two atomic layers. The resulting device achieves a record tunneling magnetoresistance (TMR) of −170.2%, consistent with simulations of ideal minority spin filtering. Temperature-dependent measurements further distinguish two dominant transport regimes, spin injection at low temperatures and spin-dependent tunneling at higher temperatures. The combined contribution of these mechanisms sustains high TMR across the full temperature range. Our findings establish a path for realizing scalable, high-performance 2D spintronic devices for applications in MRAM, spin logic, and magnetic sensing.

## Graphical abstract

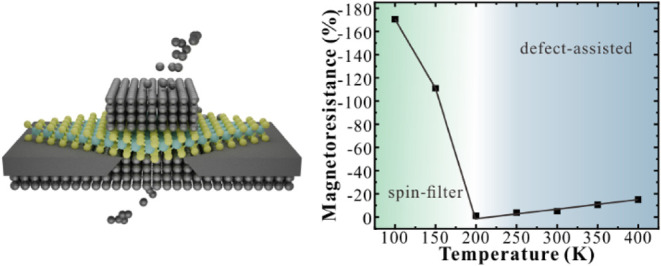

## Introduction

2D materials have emerged as a particularly promising candidate for spintronics research.^1–4^ Their atomic thickness, excellent interfacial stability, and unique electronic properties provide routes toward overcoming the limitations of traditional magnetic devices.^5–9^ Particularly, the ideal alignment between the band structure of 2D transition-metal dichalcogenides and minority-spin channels of ferromagnets such as Fe, Co, and Ni has raised the possibility of utilizing TMDCs as robust minority spin-filter barriers.^10^ The anticipated combination of efficient barrier injection and large magnetoresistance values could enhance switching contrast, readout fidelity, and energy efficiency in spintronic devices^11^ for future nonvolatile magnetic memory (MRAM),^12^ all-spin logic circuits,^13^ and high-sensitivity magnetic sensors.^14,15^

Despite significant research efforts, experimental results have consistently fallen short of theoretical expectations.^4,6^ Simulations have predicted magnetoresistance variations of 50–100% for MoS_2_-based spin-filters, but experiments have only shown values around 1%.^16–18^ Moreover, the large noise imposed by spin leakage has hindered experimental confirmation of minority spin filtering in MoS_2_.

Previous work has suggested that these issues originate from an interfacial hybridization effect.^4,19^ The proximity to Co leads to an emergence of metallic states in the MoS_2_ that have been termed “metallization”. While such interfacial interactions are also present in traditional barriers,^19–21^ the atomic thickness of 2D materials leads to their dominating influence on the electronic and magnetic response of 2D material-based spin filters. Unfortunately, these predictions have not been conclusively studied due to the simultaneous occurrence of other detrimental effects such as surface oxidation,^20–22^ contamination,^23^ and defect-induced depolarization.^1^

We here leverage a contamination-free fabrication technique to clearly demonstrate the emergence of metallization at the Co/MoS_2_ interface, study its impact on the performance of MoS_2_ spin valves, and devise a strategy to overcome it. Combined magneto-optical characterization and theoretical analysis establish the strong interaction between the ferromagnetic electrode and the 2D filter barrier. This hybridization leads to an extension of the electrode’s wave function into the MoS_2_ and decreases the effectiveness of the transmission barrier. The effect of metallization on magneto-transport was shown to be mitigated by the insertion of two atomic layers as spacers. The resulting device exhibits an increased magnetoresistance of −170.2%, significantly outperforming metalized MoS_2_ devices and previous reports. Comparison to simulation establishes the near-ideal spin filtering of this structure. Detailed analysis of the magneto-transport reveals two separate mechanisms- spin-injection and spin-dependent tunneling, that are dominant at low and high temperatures, respectively. The synergy between the two processes results in a robust and record-breaking TMR throughout the investigated temperature range. These findings represent a significant advancement toward high-performance and scalable 2D-material-based spintronic platforms for next-generation memory, logic, and sensing applications.

## Experimental Section

MoS_2_ was synthesized via chemical vapor deposition (CVD), following previous reports.^24^ Briefly, MoO_3_ (750 °C) and H_2_S (400 sccm, Ar-diluted) served as precursors for the direct growth of single-layer MoS_2_ on a Si/SiO_2_ substrate. The growth took place at 900 °C under 4 Torr, with the substrate positioned 20 cm downstream from the MoO_3_ source. To improve precursor adsorption and ensure film continuity, 0.5 sccm of O_2_ was introduced during the process. The reaction was maintained for 40 min, and the system was allowed to cool naturally to room temperature.

Raman spectroscopy was performed using a Nanobase XPER RF system with a 532 nm laser excitation source. The morphological and structural analysis was conducted via scanning electron microscopy (SEM) using an FEI Nova NanoSEM 450 FEG-SEM. To mitigate sample deformation during focused ion beam (FIB) etching, a Pt thin film was deposited as a protective layer. Additionally, selected area electron diffraction (SAED) measurements were performed using a Spectra 300 FEG-TEM operated at 300 kV. The resulting diffraction patterns were analyzed to determine the lattice constant using the Miller indices-based interplanar spacing formula for cubic crystals.^25^

For MTJ integration, MoS_2_ was transferred onto porous mechanical support containing 3 μm diameter holes using a wet transfer method following previous reports.^26–28^ A poly­(methyl methacrylate) (PMMA) layer was first spin-coated onto the MoS_2_-coated silicon substrate at 3000 rpm. The silicon layer was then etched away in a 1 M KOH solution, followed by multiple deionized water rinses to ensure the removal of residual etchants. The detached MoS_2_/PMMA membrane was carefully transferred onto the porous support. To reduce PMMA residue, the sample was subjected to thermal annealing at 400 °C under a vacuum of 8.0 × 10^–3^ Torr,^29^ followed by natural cooling inside the furnace. After suspending the MoS_2_, Co electrodes were simultaneously deposited on both sides using an e-beam evaporation system (AST E-GUN PEVA-600I) equipped with a rotational stage. The deposition was carried out under a high vacuum of 3 × 10^–6^ Torr, with the rate precisely controlled at 0.1 Å/s to ensure uniform film thickness.

To extract carrier transport features in our devices from the second derivative of the I–V characteristics, we applied a Savitzky–Golay filter with a polynomial order of 2 and a window size of 20 points. This smoothing process minimized noise amplification during numerical differentiation and allowed the reliable extraction of $\frac{d^{2}I}{dV^{2}}$ features.

The magnetic properties of our devices were examined by analyzing the hysteresis of optical responses from their metallic surfaces. A custom-built longitudinal magneto-optical Kerr effect (MOKE) system was utilized,^30^ with an in-plane external magnetic field generated by a Helmholtz coil designed to exhibit minimal remanence, ensuring precise hysteresis measurements. To evaluate magnetoresistance (MR) behavior, measurements were conducted using an AC lock-in amplifier or Keithley 2400 while applying an external magnetic field to track resistance variations. The applied bias voltages during MR characterization were set to 0.5 V for Co/monolayer MoS_2_/Co and 5 V for Co/multilayer MoS_2_/Co to investigate their respective transport properties.

To accurately reflect the value of the negative magnetoresistance observed in the Co/multilayer MoS_2_/Co, we adopted the pessimistic definition of the TMR ratio, defined as $\left(\frac{R_{AP}-R_{P}}{R_{AP}}\right)\times 100\%$ This approach better captures the relative change in resistance when R_P_ > R_AP_, and is commonly used in inverse TMR systems such as Fe_3_O_4_-based MTJs,^31^ where optimiztic definition $\left(\frac{R_{AP}-R_{P}}{R_{P}}\right)\times 100\%$ may underestimate the actual MR magnitude.

The transmission spectrum in a two-probe configuration was calculated using the nonequilibrium Green’s function (NEGF) formalism combined with LCAO–DFT as implemented in quantumATK. A GGA.PBE basis set and an SGGA exchange-correlation function were employed. Transmission was calculated on a Monkhorst–Pack grid of 7 × 7, and a density mesh cutoff of 85 hartree was chosen.

## Results and Discussion

To investigate the intrinsic response of MoS_2_ free from contaminations and interface oxidation,^23^ we leverage an uninterrupted contact deposition (UCD) process for ultraclean interfaces.^31^ First, MoS_2_ was suspended on a substrate with micrometer-sized holes.(Figure 1a) Raman spectroscopic measurement was conducted on both the suspended and supported regions, respectively (Figure 1b). For supported MoS_2_, the E_2_g and A_1_g modes exhibit a 19 cm^–1^ separation, confirming its monolayer nature.^32^ In contrast, the E_2_g mode of suspended monolayer MoS_2_ shifts to a higher frequency due to reduced strain, as this mode is particularly strain-sensitive. These Raman spectra confirm the successful suspension of MoS_2_ over microsized holes, which is a prerequisite for UCD.

**Figure 1:**
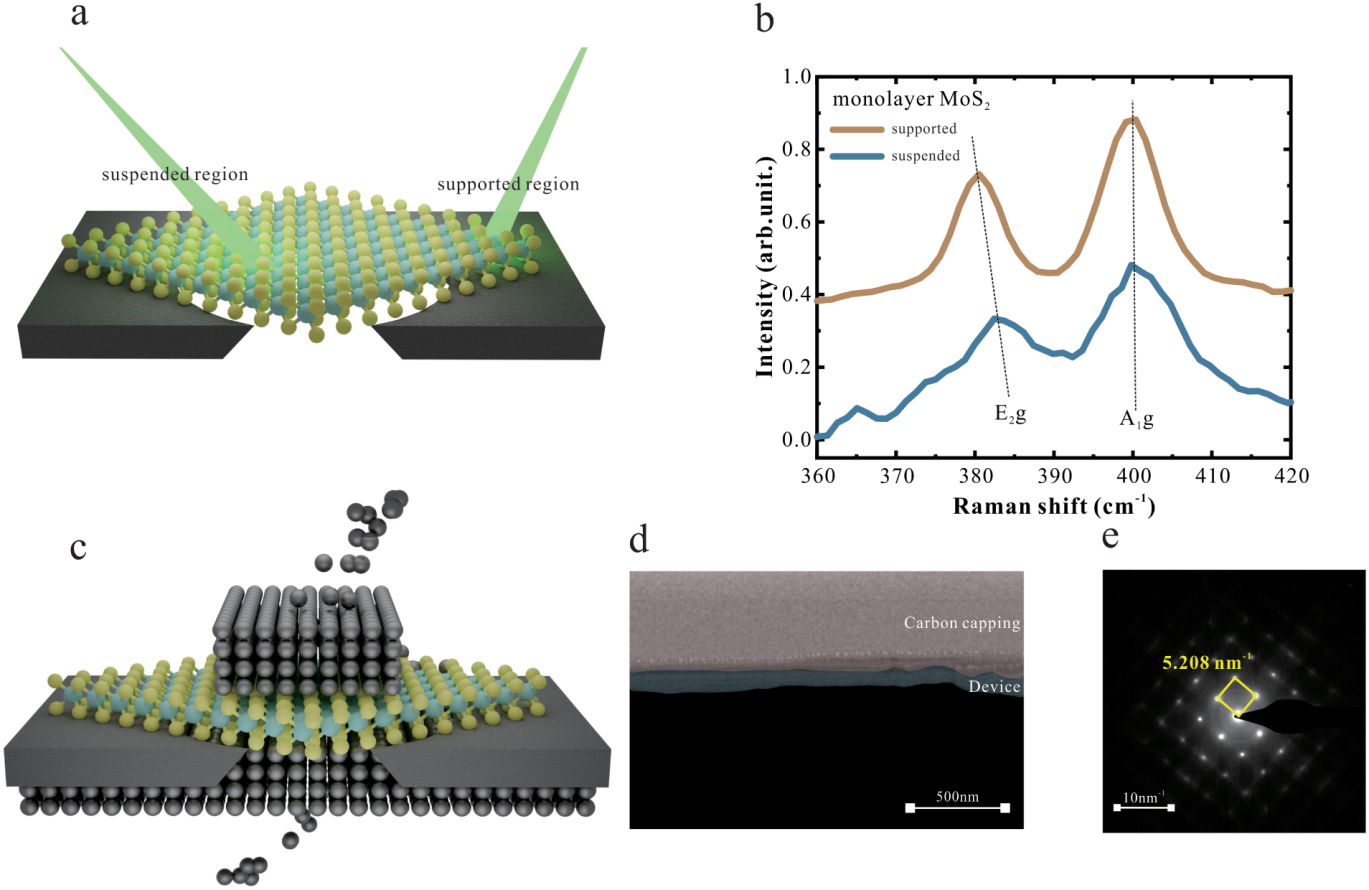
(a) Schematic of the suspended MoS_2_ structure under Raman spectroscopy. The green lights represent the probing laser in Raman spectroscopy, focusing on the suspended and supported MoS_2_ regions, respectively. (b) Raman spectra of suspended (blue line) and supported (brown line) monolayer MoS_2_. (c) Schematic of the MTJ’s structure fabricated using the UCD method. The top and bottom gray-colored atoms represent Co, while the middle layer corresponds to the MoS_2_ spacer. (d) Cross-sectional SEM image of the Co/monolayer MoS_2_/Co interface. (e) SAED pattern of the Co electrodes used in the Co/monolayer MoS_2_/Co device.

Using a modified e-beam evaporator, Cobalt electrodes were deposited on both sides of the MoS_2_ without breaking the vacuum (Figure 1c). Cross-sectional scanning electron microscopy (SEM) was conducted to inspect the interface of the Co/monolayer MoS_2_/Co structure (Figure 1d). Both the top and bottom electrodes exhibit a compact and seamless contact, with no observable gaps.

Selected area electron diffraction (SAED) performed in transmission mode through the suspended MoS_2_ film reveals sharp and well-defined diffraction spots, confirming the high crystallinity of the region under investigation (Figure 1e) (due to the large difference in thickness, the MoS_2_ diffraction cannot be resolved simultaneously). Notably, the observed square diffraction pattern is characteristic of face-centered cubic (FCC) cobalt oriented along the [001] zone axis,^33,34^ indicating the successful realization of the commonly unstable cubic Co phase.^35^ This unique structure is enabled by the stabilizing effect of MoS_2_ on the deposition process: First, previous studies have shown that metal (001) surfaces exhibit the highest adsorption energy with sulfur atoms in MoS_2._ ^36^ Second, the FCC(001) surface exhibits a significantly lower Young’s modulus (∼92 GPa) compared to FCC(111) (∼236 GPa) and hcp(0001) (∼311 GPa), implying that it is more compliant and better able to accommodate interfacial strain.^36–42^ Finally, the experimentally observed lattice constant (3.84 Å) produces a nearly commensurate 7 × 7 Co(001)/6 × 6 MoS_2_ superlattice with a residual mismatch of only ∼ 0.25%. These findings demonstrate that our UCD technique provides a novel route to achieve interfaces with highly desirable magnetic properties. (More details on the epitaxial alignment and energetics are provided in the Supporting Information.)

With the quality of the interfaces between the Co electrode and MoS_2_ established, we proceed to investigate carrier transport across them. The current–voltage characteristics exhibit a linear relationship and a significantly higher conductivity than previous Co/MoS_2_/Co structures.^43^ (Figure 2a). To explore these two unusual observations for a semiconductor/metal junction, we fit the data to a two-dimensional (2D) thermionic emission model at variable temperatures (Figure 2b).^44^ A negligible barrier height was extracted that persists across the entire voltage range examined (inset of Figure 2b), suggesting that carrier injection occurs predominantly via direct tunneling through the barrier rather than thermionic emission over it.^44^

**Figure 2:**
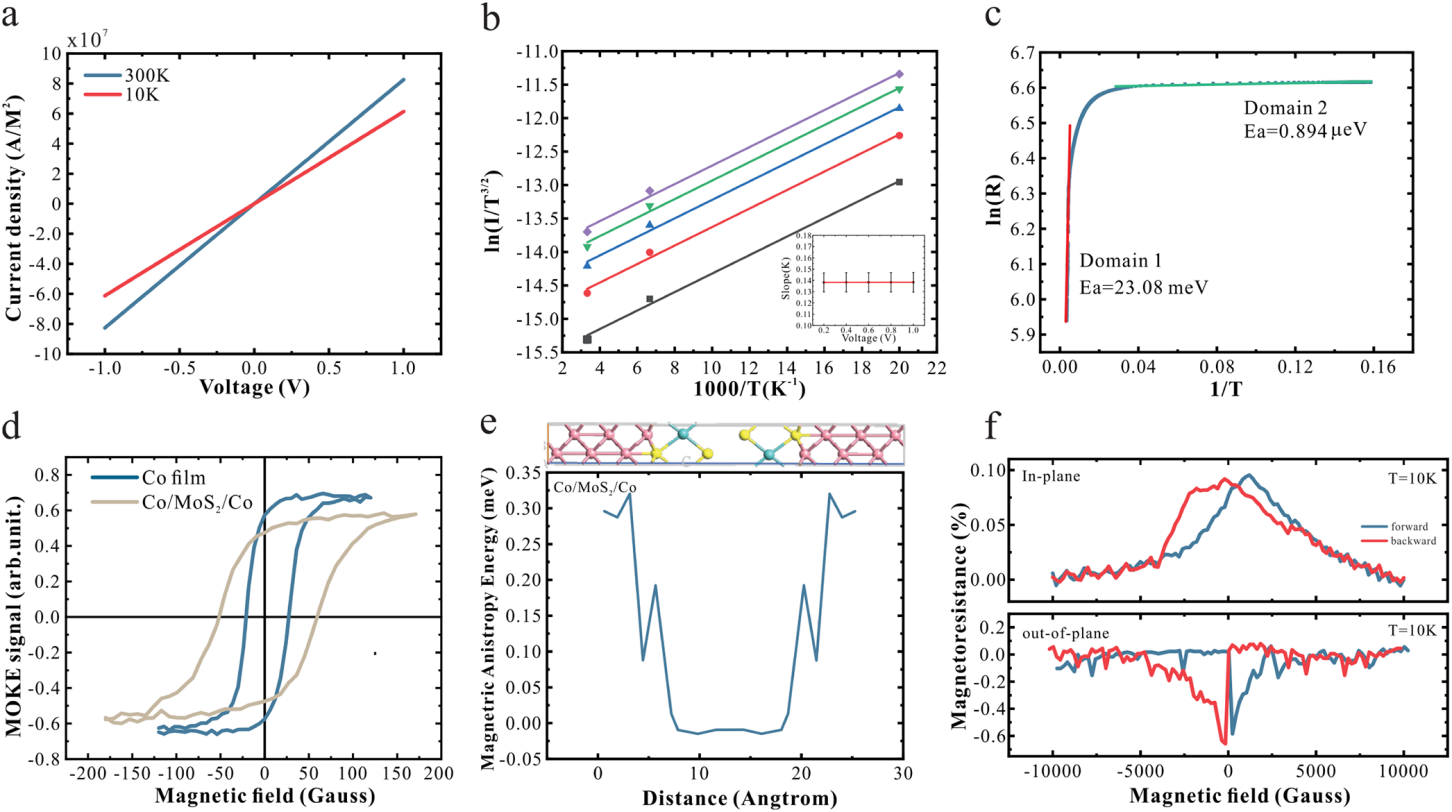
Demonstration of metalization: (a) I–V characteristics of the Co/monolayer MoS_2_/Co. (b) Arrhenius plots of ln­(I/T^3/2^) versus 1/T measured at various bias voltages. Barrier heights are estimated from the y-intercepts, as shown in the inset. Each point represents the slope extracted from the corresponding Arrhenius fit in (b) under a specific bias. (c) Arrhenius analysis based on the 2D thermionic emission model is performed on the Co/monolayer MoS_2_/Co junction to extract activation energy values. (d) MOKE hysteresis loops of the Co film (blue) and the Co/monolayer MoS_2_/Co surface (brown). (e) DFT analysis of the magnetic anisotropy energy along the lateral device. The top diagram shows the structure used in the DFT calculation. Pink atoms on both sides represent cobalt, while the blue and yellow atoms in the middle represent molybdenum and sulfur, respectively. (f) MR results at *T* = 10 K were obtained under an in-plane magnetic field (top) and an out-of-plane magnetic field (bottom).

Further insight is gained from an Arrhenius-type conduction analysis of the temperature-dependent resistance (Figure 2c).^45^ These results reveal a competition between direct tunneling and Schottky emission at temperatures above 50 K. By fitting the high-temperature regime, we extract an activation energy ***E***_***a***_ of 23.08 meV, which is consistent with previous reports on MoS_2_/metal interfaces.^46,47^

We estimate the effective thickness (*d*) of the tunneling barrier using the Wentzel–Kramers–Brillouin (WKB) approximation.^48,49^ The model considers electrons with an effective mass tunneling through a potential barrier of height *ϕ_B_* (the reported Co/MoS_2_ barrier height of 38 meV),^50^ with a thermally extracted activation energy *E*_*a*_ = 23.08 meV. The thickness is then given by

$$d=\frac{\hbar }{2\sqrt{\left(2m^{*}\phi_{B}\right)}}\ln\left(\frac{\phi_{B}}{E_{a}}\right)$$

, where *h̅* is the reduced Planck constant. Substituting the relevant values yields an effective barrier thickness of 0.353 nm, which is considerably smaller than the expected monolayer thickness of MoS_2_, typically around 0.65 nm.^51,52^

Such a marked reduction in effective barrier thickness supports the occurrence of interfacial metallization: Previous theoretical work predicted that MoS_2_ in proximity to ferromagnetic metals such as Co^52^ or Fe^53^ undergoes significant orbital hybridization, leading to the emergence of metallic states at the interface. Metallization would decrease the extent of pristine MoS_2_, resulting in a barrier width below the intrinsic MoS_2_ dimension.

The impact of the observed interfacial metallization on the magnetic properties of the Co electrodes was investigated by MOKE measurements. Compared to bare Co, introducing a MoS_2_ interface produces an increased coercivity (Figure 2d). This enhancement is particularly surprising as fcc Co is expected to exhibit a 10-fold decreased anisotropy compared to hcp Co.^54,55^ DFT calculations support this finding and reveal that SOC at the Co/MoS_2_ interface modifies the magnetic anisotropy energy (MAE) (Figure 2e). These changes suggest that the metallization enhances the magnetic anisotropy. A decreased saturation magnetization also indicates that the magnetic moment of Co is reduced, likely due to spin–orbit coupling (SOC) and interfacial hybridization.^56^

The metallization-enhanced anisotropy was further characterized by magneto-transport measurements (Figure 2 (f)). A pronounced anisotropic magnetoresistance (AMR) of |*AMR*| = 600% was observed. This value is much higher than for Co electrodes separated by Cu spacers and demonstrates the strong interfacial coupling between the ferromagnet and MoS_2_.^57^

The switching field extracted from the MR curve (Figure 2f) is significantly larger than the coercivity obtained from MOKE measurements (Figure 2d). This difference originates from the interfacial spin-pinning effect at the MoS_2_/Co contact. While MOKE primarily probes the magnetization reversal of the top Co surface, transport measurements are sensitive to the Co spins at the MoS_2_/Co interface, where hybridization and enhanced anisotropy lead to stronger pinning. Consequently, the interfacial switching requires a higher magnetic field, resulting in a nearly 10-fold increase in the critical field compared to the pristine Co layer.

Different from previous experimental work on the Co/MoS_2_ interface,^49^ our device shows negative magnetoresistance for out-of-plane magnetization (Figure 2e). This result provides the first experimental evidence that spin-filtering through MoS_2_ can suppress the majority carrier transmission at low magnetic fields. However, the magnetoresistance magnitude only reaches 0.9%, which is smaller than previous reports on MoS_2_ MTJs (see Table 1). These two observations indicate that, even in the presence of metallization, spin filtering can be achieved, but that its efficiency is severely limited.

**Table 1:** Comparison of the MR Ratio in MoS_2_-based MTJs with Previously Reported Values

Year	Structure	Maximum MR (%)	Reference
2015	Fe_3_O_4_/MoS_2_/Fe_3_O_4_	0.2 (80 K)	^60^
2015	NiFe/MoS_2_/NiFe	0.4 (10 K) 0.73 (20 K) 0.2 (240 K)	^1^
2017	NiFe/MoS_2_/Co	2 (75 K)	^49^
2017	LaSrMnO_3_/MoS_2_/NiFe	0.8 (20 K)	^3^
2018	NiFe/multilayer -MoS_2_/Co	3.2 (30 K) 0.62 (300 K)	^61^
2020	Fe_3_GeTe_2_/MoS_2_/Fe_3_GeTe_2_	3.1 (10 K)	^62^
2023	Fe_3_GeTe_2_/MoS_2_/Fe_3_GeTe_2_	15.89 (2.3 K) 0.33 (300 K)	^63^
2025	Co/MoS_2_/Co	0.09 (10 K) 0.04 (300 K)	Our work
2025	Co/multilayer MoS_2_/Co	–170.6(100 K) −5.0 (300 K) −15.0(400 K)	Our work

To mitigate the observed metallization and increase the spin-filtering effect, the MoS_2_ layer has to be decoupled from the interface. Previous work suggested that inserting a spacer at the 2D material/ferromagnet interface could decrease their interaction.^57,58^ However, the suggested thin-film deposition strategies produce relatively thick separation layers that decrease the transmission efficiency.^59^ Based on the presented results, we hypothesize that MoS_2_ layers could serve as ideal spacers. As demonstrated, metallization will produce narrow barriers that can be passed efficiently by electrons while also limiting the hybridization to the surface of the underlying spin filter. Consequently, the heterostructure consists of Co/metalized MoS_2_/pristine MoS_2_/metalized MoS_2_/Co.

Optimization of the metalized MoS_2_ thickness needs to be conducted to retain a single unperturbed MoS_2_ layer in the center. Zhang et al. (2016) predicted that interfacial hybridization is limited to two MoS_2_ layers and that the electronic properties of the third layer would be near-pristine.^10^ Following this prediction, we produce a Co/2l-metalized/Multilayer/2l-metalized/Co heterojunction. (More information on the material, such as Raman characterization and electrical measurements, is provided in the Supporting Information.)

Our approach is validated by the observation of a nonlinear *I–V* relationship (Figure 3a), indicating reduced barrier conductance and the recovery of its semiconducting behavior. Temperature-dependent resistance and Schottky-barrier measurements reveal injection barrier values of approximately 48 meV (Figure 3b). This value falls in the typical bandgap-derived activation energy of intrinsic monolayer MoS_2_ (35 ∼ 80 meV).^47^

**Figure 3:**
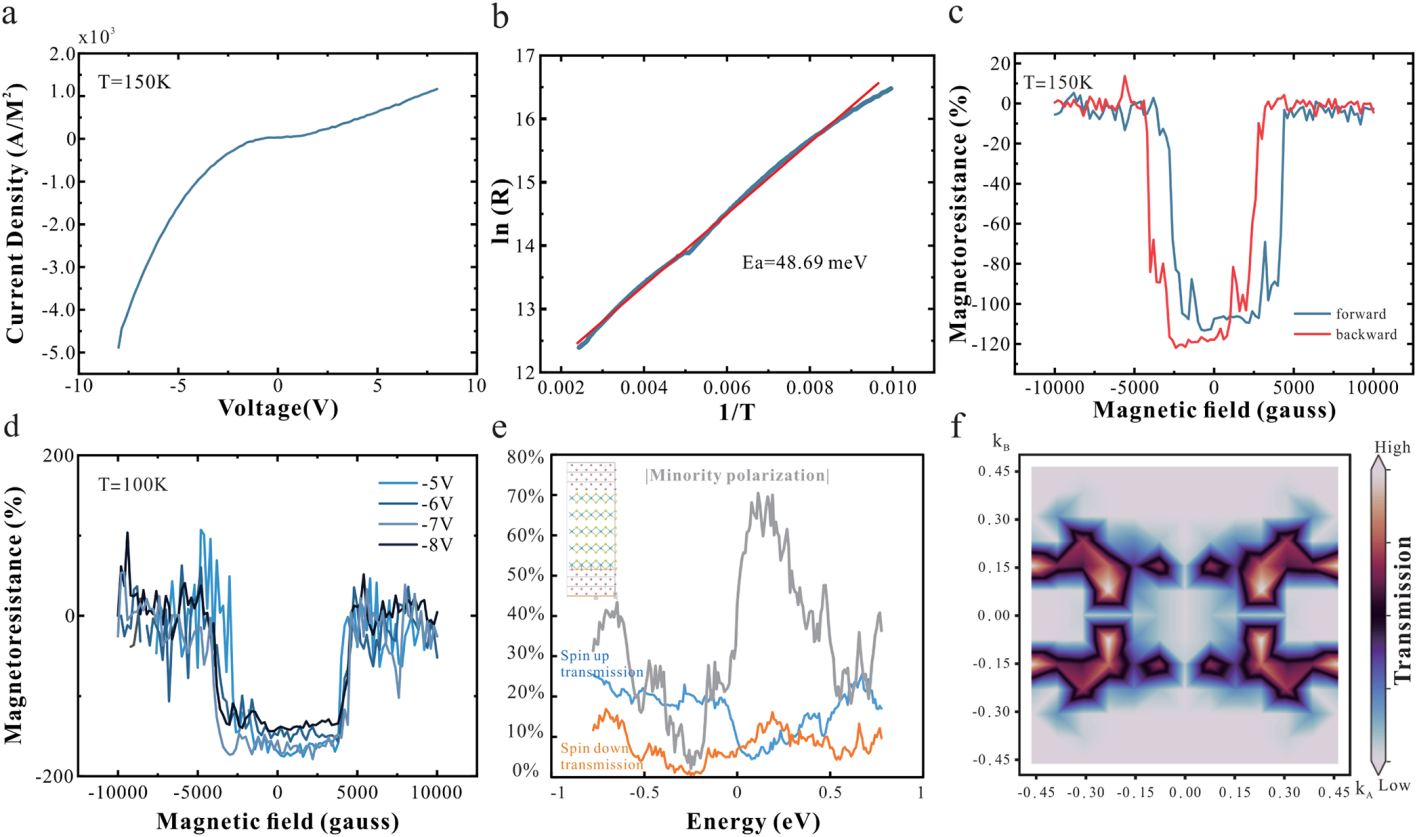
Decoupling of interfacial interaction: (a) I–V characteristics of MoS_2_ spacer device showing nonlinear behavior. (b) Arrhenius analysis based on the 2D thermionic emission model for the decoupled device. (c) MR results of the decoupled device at 150 K. (d) MR measurements under different bias voltages at 100 K. (e) Transmission spectra for minority and majority spins through the Co/MoS_2_/Co structure (inset) illustration of device structure, (f) k-resolved minority-carrier transmission at the energy of highest minority polarization (0.2 eV).

Magnetotransport measurements confirm the retention of interfacial hybridization as evidenced by a negative AMR that is comparable to the metalized MoS_2_ case (Figure 3c). However, compared to the metalized case, the decoupled MoS_2_ shows an increase in out-of-plane magnetoresistance by 284 times. An extracted value of 170.6% represents the highest reported value in 2D materials-based magnetic tunnel junctions. (Table 1)

Moreover, the negative sign of the magnetoresistance indicates that the transport relies on minority spin-filtering in MoS_2_: Tunneling-based transport between the ferromagnetic electrodes is controlled by the density of available states in the MoS_2_, which are located around the K-points. The K-point coincides with parts of the band structure in Co that exhibit higher concentrations of minority carriers, resulting in a decrease of available carriers with increasing magnetic field. The dominance of this process is confirmed by bias-dependent magnetoresistance curves (Figure 3d) that show no significant variation over a wide bias range.

From magnetoresistance measurements, a spin polarization of 68% was extracted using a simple Julliere’s model $MR=\frac{2P^{2}}{1-P^{2}}$ .^63^ This polarization is an order of magnitude higher than previous 2D materials junctions, demonstrating the effectiveness of the decoupling approach.^6^

We conduct simulations to estimate the expected spin polarization. For this purpose, we position 5 layers of MoS_2_ at the FCC (100) interface (inset Figure 3e). We establish a minority spin concentration of 70% which is in quantitative agreement with our experimental results and previous simulations on the Co(111)/MoS_2_ interface.^56^

The agreement between theoretical and experimental values indicates the near-ideal spin filtering of decoupled MoS_2_. The k-resolved transmission spectrum shows that transmission proceeds away from the Γ – point as expected for 2D materials-based spin-filtering (Figure 3f).

While multilayer MoS_2_ effectively suppresses interfacial metallization, excessive thickness introduces spin relaxation that limits polarization.^60^ In the thin limit, metallization at the interface reduces spin polarization,^53^ whereas in the thick limit, spin recombination suppresses spin transport. Therefore, a trade-off exists, and the magnetoresistance peaks at an intermediate MoS_2_ thickness where these effects are balanced, and this trade-off can be described by a modified Julliere’s model,^53,61^

$$MR=\frac{2P{\left(d\right)}^{2}\exp\left(-\frac{d}{\lambda_{s}}\right)}{1-P{\left(d\right)}^{2}\exp\left(-\frac{d}{\lambda_{s}}\right)}$$

where *P­(d)* is the thickness-dependent, intrinsic spin polarization and λ_s_ the spin diffusion length.

Finally, we investigate the high-temperature magnetoresistance due to its importance to commercial applications of spintronics. At 400 K, the magnetoresistance retains its negative value, indicating that spin-filtering is still dominant (Figure 4a). Surprisingly, however, the evolution of the magnetoresistance with temperature is not monotonic as would be expected from the proposed spin-tunneling mechanism (Figure 4b). This deviation suggests the emergence of a second spin-filtering mechanism that competes with the low-temperature mechanism.

**Figure 4:**
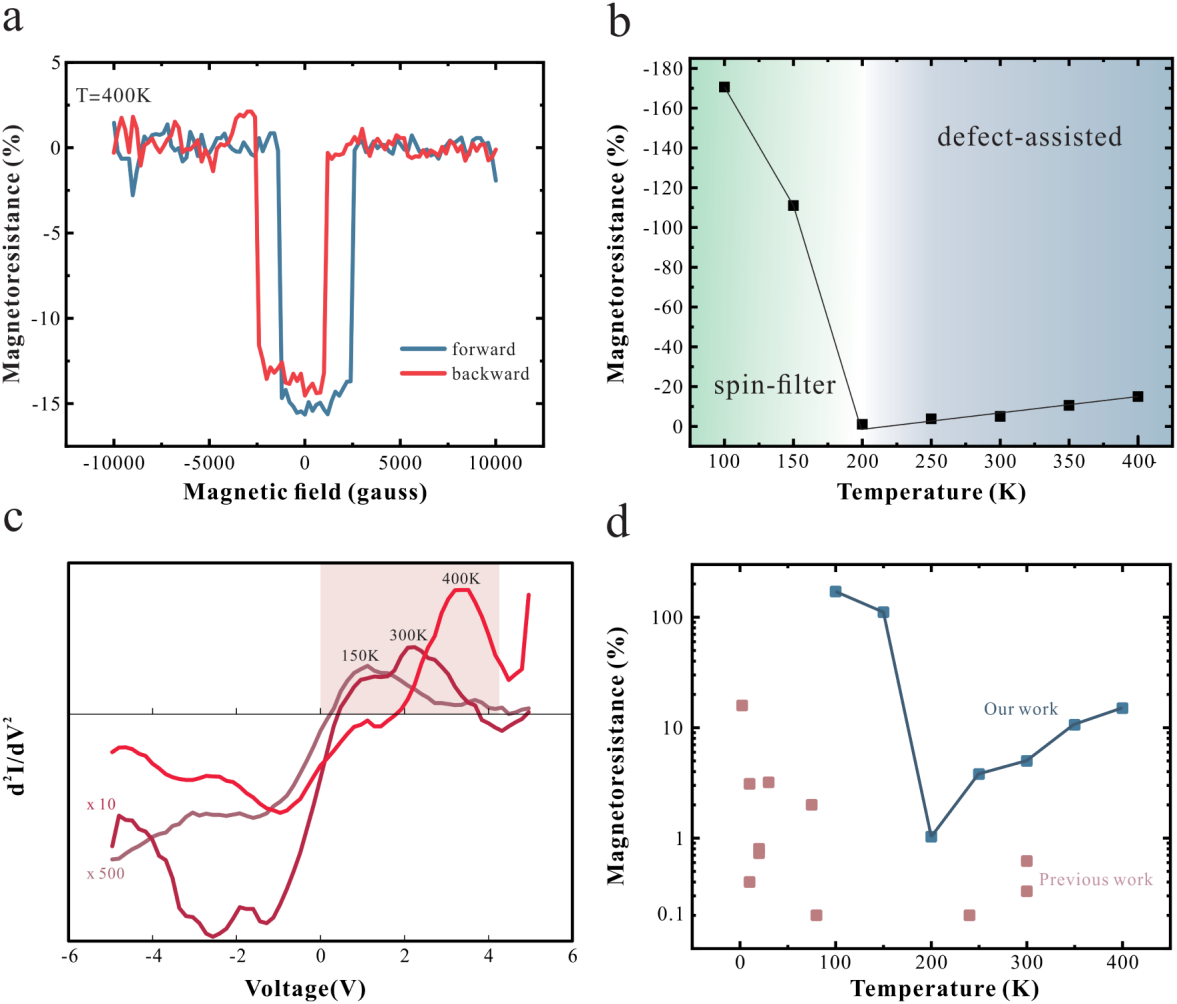
Temperature-dependence of spin-valve performance: (a) MR measurement for the decoupled device at 400 K. (b) Temperature-dependent MR results of the decoupled device. (c) The second derivative of the I–V curve with indication of the evoltuion of the feature corresponding to defect-assisted tunneling as at increasing temperature (d) Summary plot comparing our MR results (blue) with previously reported values (light purple), highlighting the record-breaking performance of our decoupled MTJ device.

To elucidate the high-temperature spin-filtering mechanism, we investigate the second derivative of the current–voltage curve, which visualizes subtle changes to the density of states.^61,64,65^ As shown in Figure 4c, a distinct spectral feature emerges that is indicative of a thermally activated transport process such as defect-assisted tunneling.^66^ Upon heating, the feature becomes more prominent, indicating the increasing accessibility of this transport channel.^67^ Our observations are consistent with previous theoretical and experimental studies showing that sulfur vacancies in multilayer MoS_2_ could support spin-polarized transmission, especially when hybridized with the band structure near high-symmetry points.^68,69^

Our results demonstrate the potential of metallization-suppressed MoS_2_ MTJ as robust and efficient spin filters for next-generation spintronics that outperform previously proposed devices in both performance and temperature windows (Figure 4d.

## Conclusion

This study identifies interfacial metallization as the key factor limiting magnetoresistance in MoS_2_-based magnetic tunnel junctions. Using uninterrupted contact deposition and detailed magnetotransport measurements, we directly observed the formation of hybridized electronic and magnetic states at the Co/MoS_2_ interface. To address this, we fabricated multilayer MoS_2_ junctions that take advantage of the limited depth of metallization, allowing the central layer to retain its pristine semiconducting properties. This structural decoupling significantly enhances spin filtering and enables a tunneling magnetoresistance of −170.6%, the highest reported among 2D-material-based spin devices. Temperature-dependent analysis further reveals two coexisting spin-filtering mechanisms: direct tunneling at low temperatures and defect-assisted transport at elevated temperatures, which together support stable operation up to 400 K. These results demonstrate a practical and scalable approach to realizing efficient, thermally stable spintronic devices using 2D materials.

## Supplementary Material



## Notes

Additional discussion of directional Young’s modulus for fcc-Co (001), (111), and hcp-Co (0001); Raman spectra of suspended multilayer MoS_2_; temperature-dependent I–V and MR measurements (PDF)
