# Supplementary material for: Observing and Suppressing Metallization in MoS2 for Near-Ideal Spin Filtering
Source: ACS Appl Mater Interfaces. 2025 Nov 19;17(48):65967–75. doi: 10.1021/acsami.5c15955 (PMC12679537; doi:10.1021/acsami.5c15955)
Supplement: Supplementary file 1 [file am5c15955_si_001.pdf]

Supporting Information

Observing and suppressing metallization in MoS_2 for near-ideal spin filtering

Ting-Chun Huang[1,2], Yu-Xin Chen[3], Yu-Lin Chen[3], Meng-Ting Wu[3], Chi-Feng Pai[4], Chiashain Chuang[3], Ya-Ping Hsieh[1], and Mario Hofmann[2]*

[1]*Institute of Atomic and Molecular Sciences, Academia Sinica, Taipei 115, Taiwan*

[2]*Department of Physics, National Taiwan University, Taipei 106, Taiwan*

[3]*Department of Electronic Engineering, Chung Yuan Christian University, Taoyuan 320, Taiwan*

[4]*Department of Materials Science and Engineering, National Taiwan University, Taipei 10617, Taiwan*

E-mail address: mario@phys.ntu.edu.tw

Calculation of Directional Young's Modulus for fcc-Co along (001), (111) and hcp-Co (0001)

To evaluate the anisotropic mechanical response of face-centered cubic cobalt (fcc-Co), the Young's modulus along the crystallographic directions (001) and (111) was derived from its elastic stiffness constants c_{11}, c_{12}, and c_{44}.[1] These constants were obtained from Brillouin light scattering measurements as reported by Gump *et al.,*[2] yielding:

c_{11}= 225 GPa, c_{12}= 160 GPa, c_{44}= 92 GPa.

In cubic crystals, the Young's modulus along a given direction can be calculated using the elastic compliance constants s_{ij}, which are the elements of the inverse of the stiffness matrix. For cubic symmetry, the relevant compliance relations are:

$$s_{11} = \frac{c_{11}+c_{12}}{(c_{11}-c_{12})(c_{11}+2c_{12})} \;, s_{12} = -\frac{c_{12}}{(c_{11}-c_{12})(c_{11}+2c_{12})},\; s_{44} = \frac{1}{c_{44}}$$

(1) For the [001] direction (normal to the (001) plane):

The directional Young's modulus is:

$$\frac{1}{E_{[001]}} = s_{11},$$

Therefore, in terms of stiffness constants:

$$E_{[001]} = \frac{(c_{11}-c_{12})(c_{11}+2c_{12})}{c_{11}+c_{12}}$$

Substituting the values:

$$E_{[001]} = \frac{(225-160)(225+2\times160)}{225+160} = \frac{65\times545}{385} \approx 91.99GPa$$

(2) For the [111] direction (normal to the (111) plane):

The compliance-based form is:

$$\frac{1}{E_{[111]}} = \frac{s_{11} + 2s_{12} + s_{44}}{3}$$

Alternatively, a more compact stiffness-based form is:

$$E_{[111]} = \frac{3(c_{11}+c_{12})c_{44}}{c_{44}+c_{11}+2c_{12}}$$

Substituting the values:

$$E_{[111]} = \frac{3(225+2\times160)\times92}{225+2\times160+92} = \frac{3\times545\times92}{637} \approx 235.86\text{GPa}$$

(3) We further compared the Young's modulus of the Co HCP (0001) structure. Based on the elastic constants reported by H.J. McSkimin[3] and the derivation provided by Newnham[4], we estimated the Young's modulus for the Co HCP (0001) orientation. [4]

From ultrasonic pulse-echo experiments at 25 °C, the five independent stiffness constants (in GPa) are reported as [3]:

$$c_{11} = 307GPa, c_{12} = 165GPa,, c_{13} = 106GPa,, c_{33} = 358GPa,, c_{44} = 80GPa$$

In a hexagonal (hcp) crystal, the longitudinal compliance along [0001] is

$$s_{33} = \frac{c_{11} + c_{12}}{(c_{11} + c_{12})c_{33} - 2c_{33}^2}$$

The Young's modulus along the c-axis is then[4]

$$E_{[0001]} = \frac{1}{s_{33}} = \frac{(c_{11} + c_{12})c_{33} - 2c_{13}^2}{c_{11} + c_{12}}$$

Plugging in the above c_{ij} :

$$E_{[0001]} = \frac{(307 + 165)358 - 2 \times 106^2}{307 + 165} = \frac{146504}{472} \approx 311GPa$$

The single-crystal Young's modulus of hcp cobalt along the [0001] direction is therefore ≈ 311 GPa.

Conclusion: Among the evaluated orientations and phases, fcc-Co (001) exhibits the lowest Young's modulus (≈ 92 GPa), compared to fcc-Co (111) (≈ 236 GPa) and hcp-

Co (0001) (≈ 311 GPa). This indicates that the (001) direction of fcc cobalt is the most compliant, offering the highest elastic deformability under uniaxial stress. Such mechanical softness is advantageous in strain engineering and heteroepitaxial growth, as it allows the Co film to better accommodate lattice mismatch and interfacial stress, particularly when interfaced with materials like MoS_2.

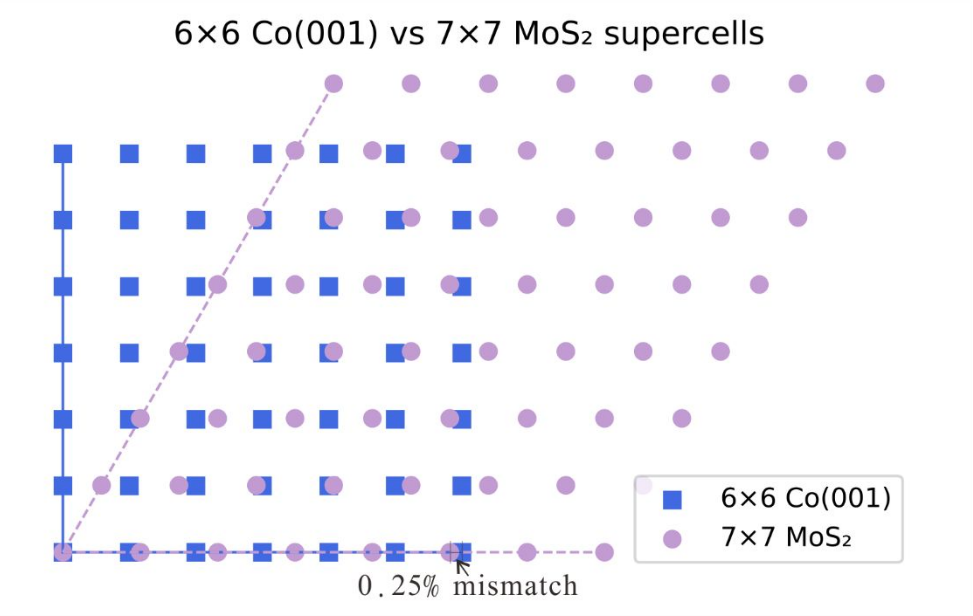

Figure S1. Schematic illustration of the two surface lattices compared in this study. Blue squares represent atomic positions in a 6 × 6 supercell of fcc-Co(001) with an in-plane nearest-neighbor spacing of 2.72 Å. Violet circles denote a 7 × 7 supercell of relaxed monolayer MoS_2 with a hexagonal lattice constant of 3.16 Å. Solid blue lines and dashed violet lines indicate the primitive lattice vectors of the Co(001) square lattice and the MoS_2 hexagonal lattice, respectively. This diagram highlights the geometric relationship between the two lattices and illustrates the near-commensurate interface formed via lattice–mismatch–induced strain accommodation.

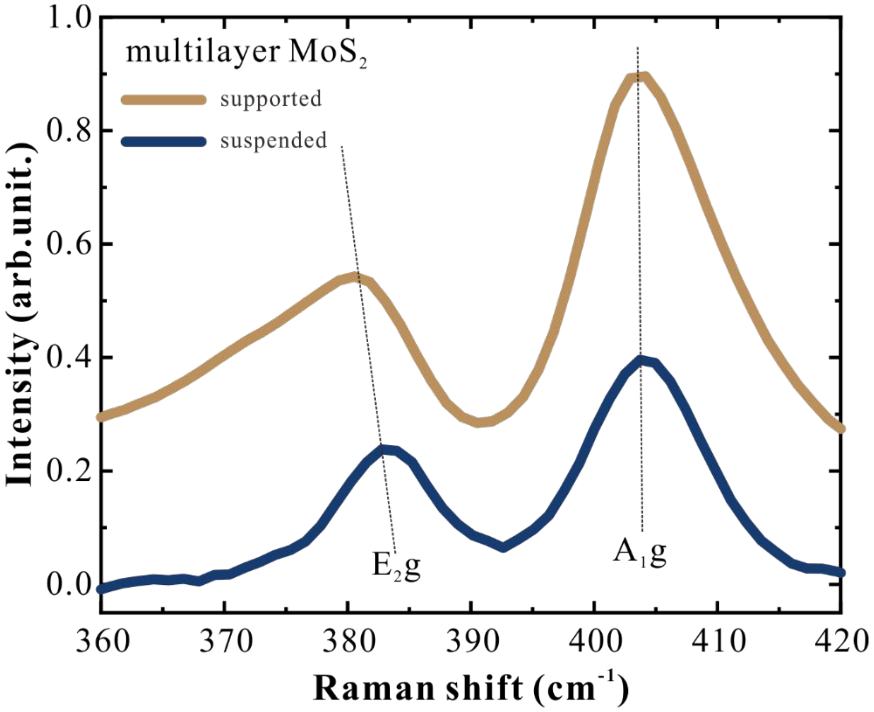

Figure S2. Raman spectra of suspended multilayer MoS_2. The E_2g and A_1g modes exhibit a separation of 25 cm^{-1}, confirming the multilayer thickness. Similar to the monolayer MoS_2 case discussed in the main text, the E_2g mode of the suspended multilayer MoS_2 shows a blue shift due to strain relaxation, as this mode is particularly sensitive to strain.

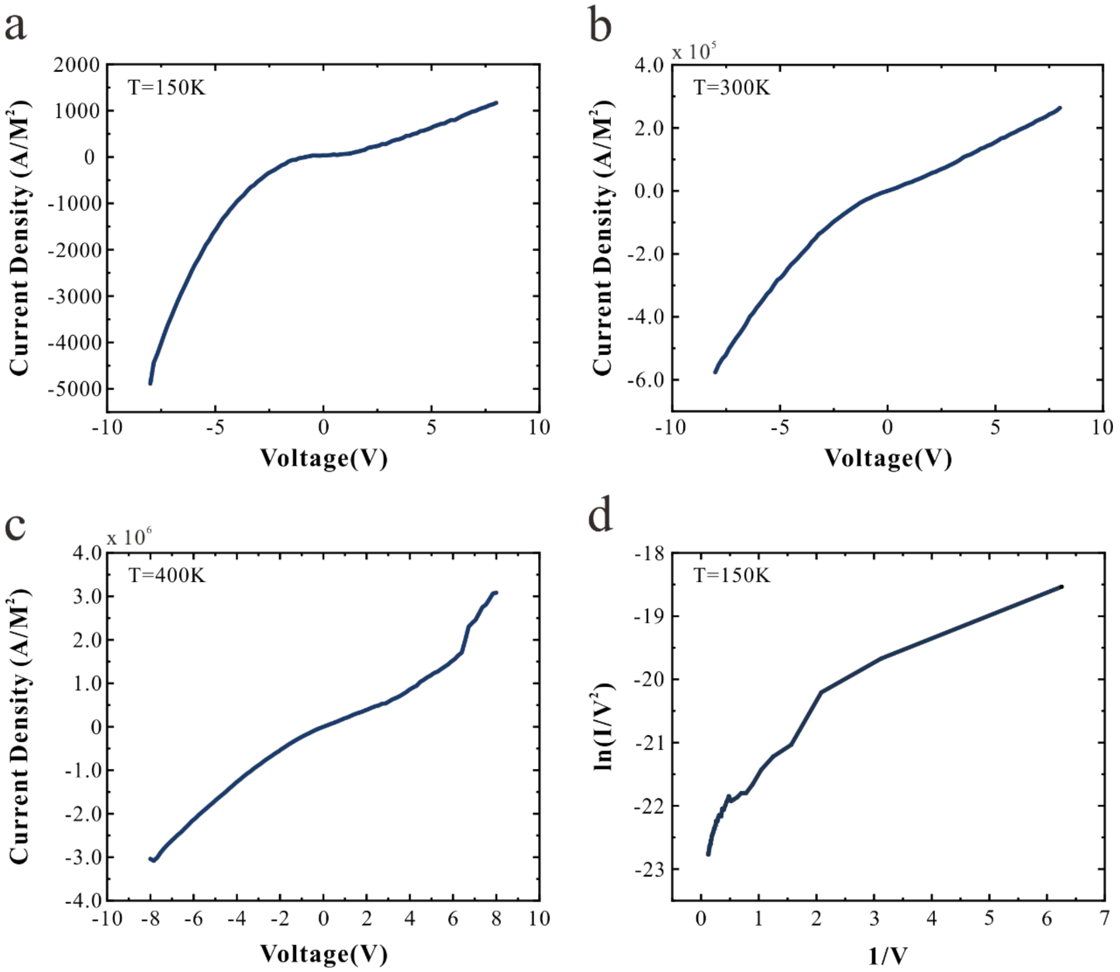

Figure S3. (a-c) *I-V* characteristics of the Co/metalized MoS_2/pristine MoS_2/metalized MoS_2/Co device at different temperatures. (d) Tunneling analysis at 150 K indicates direct tunneling behavior in the Co/metalized MoS_2/pristine MoS_2/metalized MoS_2/Co structure.

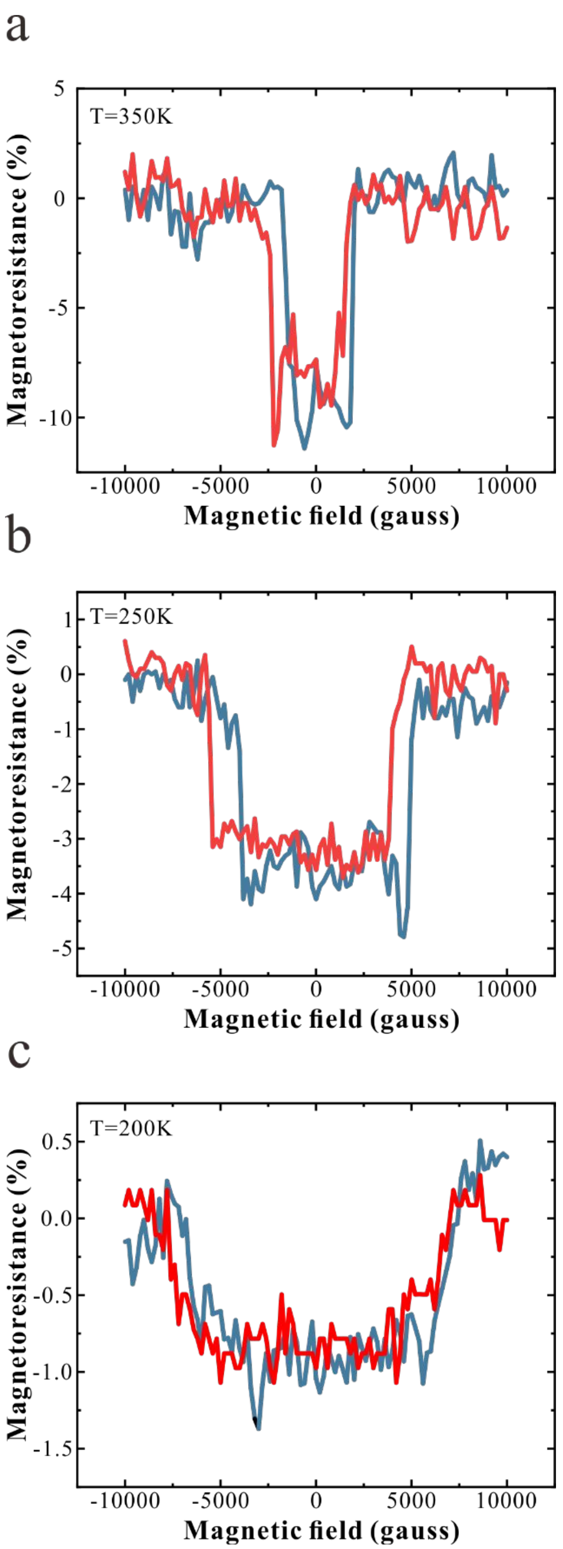

Figure S4. The pessimistic MR results of Co/metalized MoS_2/pristine MoS_2/metalized MoS_2/Co structure at (a) 350 K, (b) 250 K, and (c) 200 K.

Reference

(1) Knowles, K. M. The plane strain Young's modulus in cubic materials. *Journal of Elasticity* **2017**, *128* (2), 147-173.
(2) Gump, J.; Xia, H.; Chirita, M.; Sooryakumar, R.; Tomaz, M.; Harp, G. Elastic constants of face-centered-cubic cobalt. *Journal of Applied Physics* **1999**, *86* (11), 6005-6009.
(3) McSkimin, H. Measurement of the elastic constants of single crystal cobalt. *Journal of Applied Physics* **1955**, *26* (4), 406-409.
(4) Newnham, R. E. *Properties of materials: anisotropy, symmetry, structure*; OUP Oxford, 2004.
